# Does community pressure matter in cesarean deliveries in Bangladesh? An analysis of nationally representative surveys

**DOI:** 10.1371/journal.pone.0328162

**Published:** 2025-08-19

**Authors:** Md Rabiul Haque, Ahbab Mohammad Fazle Rabbi, Mohammad Sazzad Hasan, Fardin Araf, Md Saidul Islam

**Affiliations:** 1 Department of Population Sciences, University of Dhaka, Dhaka, Bangladesh; 2 Department of Neuroscience, Carleton University, Canada; 3 Innovision Consulting Private Limited, Bangladesh; 4 Department of Immigration and Passports, Bangladesh; Jahangirnagar University, BANGLADESH

## Abstract

Cesarean delivery plays a significant role in reducing maternal and child mortality. However, unjustified cesarean section (C-section) delivery is rising worldwide, including in Bangladesh. C-section delivery rates in Bangladesh have increased from 2.9% in 1999 to 45% in 2022, which is particularly high for first-order births (51%). This study aims to describe the prevalence and determinants of births by C-section for institutional deliveries in Bangladesh’s private and public health facilities. Data from the Bangladesh Demographic and Health Surveys (BDHS) for 2011, 2014, 2017−18, and 2022 are used in this study. Besides the common socio-economic determinants of C-sections, adequate antenatal care (ANC) visits, place of delivery (public/private), and community-level factors including level of illiteracy and prevalence of C-sections in the community were found to have a significant association. After controlling the effect of other variables, women from a community with a high prevalence of C-sections were found to be 11.68 times more likely to have a C-section in their last birth compared to women from a community with a low prevalence of C-sections. Also, the women who had their last birth in private facilities were 8.16 times more likely to have C-sections than women who delivered in public facilities. These findings suggest that the increased rate of C-sections in Bangladesh may be driven by both individual-level and provider-level factors where community pressure plays a vital role. Close monitoring, particularly in private hospitals, and community-level awareness programs about the adversity of C-sections are the proposed policy strategies to avoid unnecessary cesarean deliveries in Bangladesh.

## Introduction

The cesarean section (C-section) is a common surgical procedure performed for delivering the baby through an incision made in the mother’s abdomen and uterus [1]. A C-section delivery might be recommended in some specific cases of childbirth where it is considered safer than normal vaginal delivery due to pregnancy-related complications or life-threatening medical conditions of women and newborns [2]. For example, prolonged labor, difficult or obstructed labor, abnormal heart rate of the fetus, abnormal positions of the baby in the womb, developmental problems in the baby, multiple pregnancies like triplets, problems with the placenta or umbilical cord, and chronic health conditions of the mother are some of the medical indications of C-section. However, the alarming rise in the prevalence of C-sections has become questionable since the 2000s [3]. World Health Organization (WHO) estimated that one-third of the C-sections performed worldwide each year are without any medical indications and are labeled as “unnecessary” [4]. Although C-section is considered safer than vaginal deliveries when there are medical indications, there is also evidence indicating that maternal morbidity and mortality are higher following a C-section compared to vaginal deliveries [5,6]. As C-sections are only warranted when the risks associated with pregnancy outweigh those associated with the procedure itself [7], WHO recommended a maximum of 10–15% C-section rate regardless of the region [4,8] to encourage vaginal delivery and reduce the prevalence of unnecessary C-sections. The recommendation seems justified as a recent study showed that no additional health benefits— a reduction in mortality outcomes can be achieved if the C-section rate exceeds the WHO’s recommended threshold [9]. However, the C-section rate has continuously increased globally in both developed and developing countries including Bangladesh [10–16] even though the cost of a C-section is higher than that of normal vaginal delivery [17]. Therefore, the rising rate of C-sections may put further pressure on the persisting high out-of-pocket expenditure burden in a country like Bangladesh [18].

One of the most common medical indication for C-sections is found to be having a previous C-section delivery with no other valid indications [19]. According to the American College of Obstetricians and Gynaecologists, a previous C-section is not a recommended indication for delivery by C-section if there are no other obstetric emergencies are present [20]. WHO encouraged vaginal deliveries following a C-section wherever there is access to emergency surgical intervention [8]. Although C-section rates are a reflection of both medical offers and social demand [3,21], opting for a surgical delivery is not a simple choice [22]. A range of non-medical factors are becoming popular gradually and triggering unnecessary C-sections [15,22] that may lead to detrimental effects on the morbidity and mortality of mothers, newborns, and infants [23]. Non-medical reasons for C-sections include the convenience of physicians—such as the availability of skilled staff and hospital facilities, as well as whether the delivery takes place in a private or public institution [7,15,23–25]. Additionally, maternal requests, which may stem from a desire to avoid pain or negative neonatal outcomes in favor of a planned C-section delivery, also play a role [21,26,27], or socioeconomic and cultural factors [16,28,29]. The impact of social demand through community influence is also subject to analysis for such a high prevalence of C-sections all over the world [15].

Bangladesh Demographic and Health Survey (BDHS) 2022 showed that the C-section rate has increased from 24% in 2014 to 34% in 2017–18 and 45% in 2022 [14]. This rate is above the WHO recommended level since 2011 (15%). Therefore, gaining more insight into the factors and underlying sociocultural norms driving the rise in C-section rates is crucial. According to BDHS-2022, 45% of live births were delivered via C-section in Bangladesh over the past three years; however, among institutional births, it rose to 70.4% [14], making it the highest rate among nine South and Southeast Asian nations, including Vietnam, Pakistan, India, Nepal, and the Maldives [16,30]. This is a matter of concern as most of the deliveries now take place in private or public institutions. Several contributing factors to higher rates of C-sections were found in Bangladesh by earlier studies. Hospital-based studies, for example, reported that both medical such as a previous history of C-section, fetal distress, cephalo-pelvic disruption, and prolonged labor pain, and non-medical factors such as improved socioeconomic status, early age pregnancy, patient preference, and doctor advice caused an increased rate of C-section [21,31,32]. C-section deliveries were more common among women who were educated, employed, lived in urban areas, belonged to wealthier families, had four or more prenatal visits, gave birth in private hospitals, had better access to media and medical facilities, and whose husbands had more education than their respective counterparts, according to recent studies conducted based on national level data [15,16,33–39]. These studies considered all births as samples even those that occurred at home and also found regional variation in C-sections, with a greater concentration in the Dhaka, Rajshahi, and Khulna divisions. However, none of the previous studies examined the effects of community-level illiteracy and community-level prevalence of C-sections on the subsequent deliveries using the most recent wave of BDHS data, 2022. Therefore, this study has focused on institutional deliveries only to provide a better understanding of the prevalence of C-sections among women in Bangladesh by comparing their socioeconomic factors and community-level indicators along with the common demographic variables. This study will help us understand why the cesarean birth rate is increasing and what interventions are required to limit the C-section epidemic, particularly in Bangladesh.

### Data and methods

In this study, we utilized data from the Bangladesh Demographic Health Surveys (BDHS). Conducted nationally and cross-sectionally, the DHS surveys are representative of health and family planning in over 85 low- and middle-income countries. Authorized by the National Institute for Population, Research, and Training (NIPORT) of the Ministry of Health and Welfare in Bangladesh, DHS surveys are made possible through USAID’s long-standing engagement [40]. To depict the prevalence of cesarean births in Bangladesh, we considered seven consecutive DHS surveys (1999−2000, 2004, 2007, 2011, 2014, 2017−18, and 2022). BDHS 1993−94 and 1996−96 were excluded due to the unavailability of the main outcome variable. For examining the determinants of cesarean births in institutional deliveries, we used the last four consecutive surveys (2011, 2014, 2017−18, and 2022). We used the latest four waves of BDHS data instead of earlier surveys (1999–2000, 2004, 2007) for several reasons. First, the most substantial rise in C-section prevalence has occurred in recent years, and current government and public health concerns are focused on this recent trend. Second, earlier datasets had a reduced number of administrative divisions (e.g., only four divisions in some cases, increasing to six in 2017–18), making regional comparisons inconsistent. Third, the earlier surveys had higher levels of missing data, particularly for maternal healthcare variables, which limited the comparability and reliability of trend analyses.The BDHS employs a two-stage stratified sample of households, encompassing strata for rural and urban areas to collect data from all seven divisions in the country. For detailed sampling procedures, refer to other BDHS publications [14,40–42]. BDHS 2011, BDHS 2014, BDHS 2017−18, and BDHS 2022 included 17,749, 17,863, 20,127, and 30,078 ever-married women of reproductive age, respectively. The BDHS considers the last birth that occurred in the corresponding survey preceding 36 months to estimate the indicators of maternal health care. Hence, the corresponding number of (last) births in BDHS 2011, 2014, 2017−18 and 2022 was 4,779, 4,626, 5,045, and 5,019 respectively. Among them, 1,367, 1,784, 2,317, and 3,209 births occurred in institutional facilities, and these specific births are considered as the study sample.

### Variables considered in this study

#### Response variable: Caesarian birth.

The study’s outcome variable is whether women had a cesarean section (C-section) during their last birth (yes, no). In the DHS survey, women are asked about all births that occurred in the three years preceding the survey with the question: ‘Was (NAME) delivered by cesarean, that is, did they cut your belly open to take the baby?’ If the woman answers yes, the corresponding response is considered a birth that occurred by C-section; otherwise, it is considered a no.

#### Independent variables.

We considered three different groups of independent variables to explain the occurrence of cesarean births. Among individual-level variables, we assessed women’s current age, whether the last birth occurred during teenage years (teen pregnancy or not), the birth order of the index child, the number of antenatal care (ANC) visits before the index birth (less than 4 visits and 4 or more visits), the place of delivery for the index child (public and private facility), the education level of women (primary or lower and secondary or higher), the employment status of women during the survey (unemployed and employed), the husband’s education level (primary or lower and secondary or higher), the religion of women (Muslim and others), and mass media exposure status (joint exposure status for newspaper, radio, and television).

Among family-level variables, we considered only the wealth quintile (poorer, poor, middle class, rich, richer). The wealth index variable in DHS data is constructed using principal component analysis (PCA) based on household ownership of selected assets (e.g., television, bicycle), housing characteristics (e.g., floor material), and access to services (e.g., water, sanitation). Households are ranked by their PCA scores and divided into quintiles (poorest to richest) within each survey [43]. The four explanatory variables that were considered as community-level variables are the administrative divisions, place of residence (urban, rural), community-level illiteracy, and community-level prevalence of C-section for the last birth. Before BDHS 2017−18, Mymensingh was not a division and was included in the Dhaka division for geographical location. Hence, we also considered Mymensingh inside the Dhaka division for BDHS 2017−18 and BDHS 2022 to harmonize the data.

The last two community-level variables are estimated using participants’ individual-level data on education at the PSU (considered as a community) and community-level prevalence of C-sections for the last birth in that PSU. Following previous literature, aggregate values of community-level illiteracy are measured by the proportion of women with a minimum primary level of education obtained from data on their level of education, categorized as “low” for less than 25%, “moderate” for 25%−50%, and “high” if the poverty level is more than 50% [44]. A similar measurement is used for the community-level prevalence of C-sections for the last birth, also constructed for those births only that occurred in institutional facilities.

### Statistical analysis

Chi-square tests have been performed to check the bivariate relationship of C-section and other background variables. The net effect of each independent variable after adjusting for other covariates was examined using binary logistic regression models where the dependent variable was the experience of having a C-section in the last birth (yes or no). All coefficients were reported with odds ratio (adjusted) and 95% confidence intervals. Sampling weights were applied in all analyses. To address the multicollinearity issues in regression analysis, we considered VIF and removed variables from regression models for which VIF was higher than 3.5. Analysis was performed using IBM SPSS version 26.

### Ethical statement

Human subjects were used in this study, however, since it is secondary data analysis, the authors were not directly involved in the data-gathering process. In Bangladesh, NIPORT and ICF International conducted the survey, and all interviews were performed by trained professional personnel. Before starting the interview, verbal consent from each respondent was taken after reading the informed consent statement. Both the Bangladesh Medical Research Council (BMRC) ethics committee and the ICF Institutional Review Board following the ethical guidelines of the Helsinki Declaration of 1964 approved the 2022 BDHS protocol.

## Results

**Fig 1:**
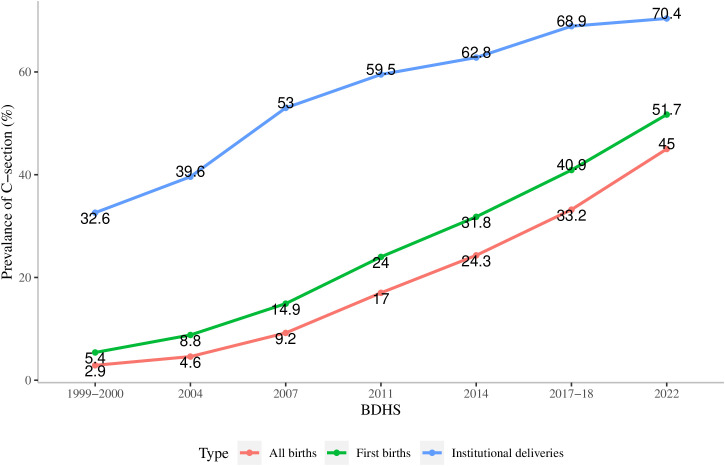
Trend of C-sections in Bangladesh from 1999-2000 to 2022 (BDHS).

The trend of cesarean births in Bangladesh is presented in Fig 1. These estimates are based on cesarean births that occurred in the survey preceding the past 3 years. The prevalence of cesarean births is considered for three different groups: all births, first births (which are the first child of a woman), and births that occurred in the institutional facilities. From 1999–2000 to 2022, the prevalence of C-section deliveries in Bangladesh rose sharply across all categories. The increase was most dramatic for first births (from 5.4% to 51.7%) and all births (from 2.9% to 45%), while institutional deliveries consistently had the highest rates, climbing from 32.6% to 70.4%. The most significant rise occurred between 2007 and 2011, particularly for first births, which jumped by 9.1 percentage points, indicating a rapid uptake of C-sections during that period.

The association between different background variables and the occurrence of C-section is presented in Table 1. The significance of these associations is assessed using the chi-square statistic, and the corresponding p-values are provided next to the name of each variable. Among the community-level variables, the community-level prevalence of C-sections in the last birth identified a highly significant relationship with C-sections in all four BDHSs. Women from communities with a higher level of prevalence of C-sections are found to have a higher prevalence of C-sections for the last birth (from 77.3% in 2011 to 87.5% in 2022). However, less impact of community-level illiteracy is observed on the prevalence of C-section. An increasing trend was observed among women from a highly illiterate community until 2017−2018, however, it drastically dropped from 52.9% in 2011 to 48.1% in BDHS 2022. The administrative division also appears a highly significant association with C-sections. The highest prevalence of C-sections is observed in the Dhaka division in all DHSs. Place of residence shows a significant association with C-sections in BDHS 2014 and 2017−18, with an increasing trend observed for both urban, from 58.4% in 2011 to 74.3%, and rural, from 60.2% to 68.6% for the same period, areas.

**Table 1:** Percentage of women who had cesarean births (BDHS 2011, 2014, 2017−18 & 2022) by selected background characteristics.

Background characteristics	BDHS (no. and percentages of observations)			
	2011 (1367)	2014 (1784)	2017−18 (2317)	2022 (3209)
Community-level prevalence of C-sections in last birth	p < 0.001	p < 0.001	p < 0.001	p < 0.001
Low (25% or less)	49.5	43.4	40	35.7
Moderate (between 25–50%)	71.5	67.8	70.6	62.9
High (More than 50%)	77.3	85.1	84.3	87.5
Community level illiteracy	p = 0.004	p < 0.001	p = 0.039	p < 0.001
Low (25% or less)	65.7	70.5	71.5	73.2
Moderate (between 25–50%)	60	62.1	68.2	68.6
High (More than 50%)	52.9	54.9	63.8	48.1
Division	p < 0.001	p < 0.001	p < 0.001	p < 0.001
Barisal	60.7	60.5	65.4	72
Chittagong	55.4	54.3	59.9	53
Dhaka	68	73.4	76.8	76.6
Khulna	58.7	62.3	70.9	80.4
Rajshahi	58.5	58.3	69.2	77.7
Rangpur	43.6	51	61.9	70.1
Sylhet	58.7	51.5	59.6	53.9
Place of residence	p = 0.503	p < 0.001	p < 0.001	p = 0.01
Urban	58.4	68.5	73.8	74.3
Rural	60.2	59.1	66.5	68.6
Age of mothers	p < 0.001	p = 0.004	p = 0.002	p = 0.708
15-19	46.1	58.8	62.5	70.4
20-24	61.2	61.1	69	68.6
25-29	62.4	63.8	68.2	71.5
30-34	69	69.3	75.5	70.9
35-39	61.2	75.3	78.3	72.3
40 or older	66.7	38.9	64	77.1
Age at first birth	p < 0.001	p < 0.001	p < 0.001	p < 0.001
Not teen pregnancy	70.5	72.5	76.8	74.6
Teen pregnancy	52.3	56.7	63.9	67.1
Birth order	p = 0.062	p = 0.003	p = 0.003	p < 0.001
1	60.7	66	68.8	72.1
2	61.6	62.4	72.4	74.2
≥3	53.3	55.4	63.1	60.6
Number of ANC visits	p = 0.003	p < 0.001	p < 0.001	p < 0.001
≤3	55.7	56.5	60.3	67.2
≥4	63.6	69.9	74.4	73.5
Place of delivery	p < 0.001	p < 0.001	p < 0.001	p < 0.001
Public facilities	43.1	38.7	36.1	36.2
Private facilities	70.9	75.2	83.7	82.7
Education of mother	p < 0.001	p < 0.001	p = 0.001	p < 0.001
Primary or lower	47.6	47	62.4	62.1
Secondary or higher	63.6	68.2	70.5	72.4
Employment status of mother	p = 0.253	p = 0.169	p < 0.001	p = 0.258
Unemployed	59.1	63.6	71.2	70.7
Employed	64.6	59.6	63.5	69.3
Husband’s education level	p < 0.001	p < 0.001	p < 0.001	p < 0.001
Primary or lower	47.4	50.3	62.8	63.9
Secondary or higher	65.9	69.6	71.9	73.8
Religion	p = 0.373	p = 0.452	p = 0.079	p = 0.743
Muslim	59.9	63.1	69.4	70.5
Others	56.4	60.1	63.9	69.5
Mass media exposure (Newspaper/Radio/Television)	p < 0.001	p < 0.001	p < 0.001	p < 0.001
Unexposed	46.4	52.8	56.5	65
Exposed	62.3	65.5	72.4	73.3
Wealth quintile	p < 0.001	p < 0.001	p < 0.001	p < 0.001
Poorest	27.1	47.1	51.7	56.6
Poorer	55.5	44.9	62.5	67.8
Middle	58.6	56.7	65.9	69.9
Richer	58.1	63.9	68.5	71.7
Richest	68.9	74.6	80.6	78.5

Note: “Division”, Mymensingh merged with Dhaka for BDHS 2017−18 and 2022 to harmonize the data with BDHS 2014 & 2011.

Among individual-level variables, the age of the women is found to have a significant association with having a C-section at the last birth, with a higher prevalence observed for women aged 20–40 in all four BDHSs. Similar findings are observed for the age at first birth, which shows a highly significant association with C-sections. C-section delivery rates increased more among women who had teenage pregnancy, from 52.3% in 211 to 67.1% in 2022. Birth order also exhibits a significant association with C-sections, and the prevalence of C-sections for each birth shows an increasing trend from 2011 to 2022. The number of ANC visits and the place of delivery also show a significant association with C-section. The prevalence of C-sections has notably increased for private facilities over time, from 70.9% to 82.7% between 2011 and 2022, while it has shown a decreasing trend for public facilities, from 43.1% to 36.2% for the same period. Among various factors, there is a significant association between C-sections and the education levels of both women and their husbands, as well as exposure to mass media. However, women with primary or below level education experienced a greater prevalence of C-sections between 2011 (47.4%) and 2022 (62.1%) than women with a secondary or higher level of education, from 63.6% to 72.4% for the same period. Similarly, C-section delivery rates between 2011 and 2022 have increased less among women with media exposure, from 62.3% to 73.3%, than among women with not exposed to media, from 46.4% to 65.0%. Additionally, household attributes—specifically wealth quintiles—show a significant association with C-sections, with the wealthiest group experiencing the highest prevalence, from 68.9% in 2011 to 78.5% in 2022. Interestingly, the poorest group shows the highest increasing trend over time in the occurrence of C-sections for their most recent births, from 27.1% in 2011 to 56.6% in 2022. To assess temporal changes in C-section prevalence, we compared rates across background characteristics using the four most recent BDHS survey waves. Due to model instability and inconsistent associations when time was included as a covariate in pooled regression models, we instead present a summary table (Table A1, Appendix) showing the relative changes in C-section prevalence over time, using 2022 as the reference year (assumed peak). This approach highlights evolving patterns and inequalities, with p-values indicating statistically significant changes over time. The detailed results are provided for interested readers in Table A1 in the appendix.

Findings from the binary logistic regression are presented in Table 2. To compare and contrast the determinants of C-section between the BDHSs, all variables considered in bivariate analyses are also included in the regression analysis. The adjusted odds ratios are presented with a 95% confidence interval. We considered four models during the analysis, one for each BDHS. The first model contains only the community-level variables (illiteracy and prevalence of C-section), the second model additionally contains the individual-level variables; the third model contains all the previous variables and family-level variables, and the full model contains all the variables. However, to reduce the volume of the results, only the full models are presented in Table 2.

**Table 2:** Determinants of having C-section among women who delivered their babies at a health center in Bangladesh (BDHS 2011, 2014, 2017−18 & 2022).

Variables	BDHS 2011	BDHS 2014	BDHS 2017−18	BDHS 2022
Community-level prevalence of C-sections in the last birth				
Low (25% or less)	ref	ref	ref	ref
Moderate (25–50%)	2.484*** (1.84,3.35)	2.749*** (2.08,3.64)	3.104*** (2.35,4.10)	2.914*** (2.262, 3.75)
High (More than 50%)	2.989*** (1.85,4.83)	8.032***(5.48,11.77)	4.976*** (3.56,6.95)	11.679*** (8.71,15.67)
Community level illiteracy				
Low (25% or less)	ref	ref	ref	ref
Moderate (25–50%)	0.987 (0.70,1.39)	1.671** (1.23,2.28)	1.192 (0.91,1.56)	1.183 (0.957, 1.46)
High (More than 50%)	1.164 (0.77,1.77)	2.751*** (1.83,4.14)	1.498* (1.00,2.24)	1.349 (0.789, 2.31)
Division				
Barisal	*ref*	*ref*	*ref*	*ref*
Chittagong	0.977 (0.51,1.89)	0.752 (0.43,1.33)	0.490* (0.29,0.85)	0.585* (0.367, 0.933)
Dhaka	1.680 (0.87,3.25)	1.085 (0.61,1.93)	0.988 (0.58,1.70)	0.989 (0.618, 1.582)
Khulna	0.966 (0.49,1.91)	0.750 (0.40,1.41)	0.858 (0.48,1.55)	0.677 (0.402, 1.139)
Rajshahi	1.110 (0.56,2.22)	0.822 (0.44,1.55)	0.884 (0.49,1.59)	0.705 (0.419, 1.189)
Rangpur	0.774 (0.38,1.60)	0.696 (0.36,1.35)	0.624 (0.34,1.15)	0.823 (0.490, 1.382)
Sylhet	1.075 (0.48,2.42)	0.932 (0.44, 1.96)	1.017 (0.52,1.97)	0.641 (0.358, 1.147)
Place of residence				
Urban	*ref*	*ref*	*ref*	*ref*
Rural	2.673*** (1.94,3.68)	1.286 (0.97,1.70)	1.058 (0.80,1.39)	1.215 (0.960, 1.537)
Age				
15-19	*ref*	*ref*	*ref*	*ref*
20-24	1.460 (0.99,2.15)	0.994 (0.69,1.44)	1.022 (0.72,1.44)	0.919 (0.661, 1.28)
25-29	1.624 (0.98,2.68)	1.619 (0.99,2.66)	0.958 (0.61,1.51)	1.049 (0.649, 1.59)
30-34	2.320* (1.21,4.45)	2.133* (1.16,3.92)	1.433 (0.82,2.50)	1.163 (0.709, 1.91)
35-39	1.933 (0.81,4.60)	2.860* (1.26,6.51)	2.112 (0.96,4.66)	1.221 (0.666, 2.24)
40-44	2.680 (0.72,9.94)	0.622 (0.18,2.13)	0.763 (0.25,2.37)	1.635 (0.561, 4.77)
Age at first birth				
Not teen pregnancy	*ref*	*ref*	*ref*	*ref*
Teen pregnancy	0.764 (0.55,1.06)	0.777 (0.57,1.05)	0.734* (0.55,0.98)	0.743* (0.583, 0.95)
Birth order				
1	*ref*	*ref*	*ref*	*ref*
2	0.922 (0.66,1.30)	0.668* (0.48,0.94)	1.171 (0.86,1.59)	1.038 (0.790, 1.36)
≥3	0.661 (0.41,1.08)	0.555* (0.35,0.89)	0.753 (0.48,1.17)	0.600** (0.411, 0.87)
Number of ANC visits				
≤3	*ref*	*ref*	*ref*	*ref*
≥4	0.978 (0.75,1.28)	1.162 (0.91.1.48)	1.585*** (1.26,2.00)	1.119 (0.918, 1.37)
Place of delivery				
Public facilities	*ref*	*ref*	*ref*	*ref*
Private facilities	2.858*** (2.23,3.67)	4.279*** (3.36,5.45)	7.975** (6.35,10.02)	8.160*** (6.658, 10.0)
Education of mother				
Primary or lower	*ref*	*ref*	*ref*	*ref*
Secondary or higher	1.287 (0.93,1.79)	1.842*** (1.37,2.48)	0.879 (0.65,1.19)	1.238 (0.952, 1.61)
Employment status of mother				
Unemployed	*ref*	*ref*	*Ref*	*ref*
Employed	0.870 (0.55,1.38)	0.893 (0.66,1.20)	0.736* (0.57,0.94)	0.944 (0.738, 1.21)
Husband’s education level				
Primary or lower	*ref*	*ref*	*ref*	*ref*
Secondary or higher	1.404* (1.04,1.89)	1.227 (0.925,1.63)	0.872 (0.67,1.14)	1.038 (0.829, 1.30)
Religion				
Muslim	*ref*	*ref*	*ref*	*ref*
Others	0.804 (0.55,1.17)	1.111 (0.738,1.67)	0.778 (0.55,1.11)	0.851 (0.611, 1.19)
Mass media exposure				
Unexposed	*ref*	*ref*	*ref*	*ref*
Exposed	1.290 (0.90,1.84)	0.709* (0.51,0.98)	1.300* (0.99,1.71)	0.939 (0.760, 1.16)
Wealth quintile				
Poorest	*ref*	*ref*	*ref*	*ref*
Poorer	2.171** (1.21,3.90)	0.856 (0.52,1.40)	1.292 (0.8,1.96)	1.338 (0.95, 1.88)
Middle	1.983* (1.13,3.50)	1.130 (0.69,1.85)	1.223 (0.80,1.87)	1.206 (0.85, 1.71)
Richer	1.737 (0.98,3.07)	1.082 (0.65,1.80)	1.179 (0.76,1.83)	1.085 (0.76, 1.55)
Richest	2.378** (1.29,4.39)	1.194 (0.69,2.07)	1.805* (1.11,2.94)	1.279 (0.87, 1.89)
Constant	0.065***	0.132***	0.175***	0.133***
−2 Log likelihood	1534.83	1809.9	2066.02	2730.64
Cox & Snell R Square	0.191	0.252	0.282	0.299
Nagelkerke R Square	0.258	0.344	0.397	0.425

Note: ***p < 0.001, **p < 0.01, *p < 0.05.

After controlling for the effect of other variables, our study found the strongest and most significant correlation between the occurrence of C-sections in the last birth and the community-level prevalence of C-sections, as shown in all four models. The odds of C-sections increase with increased prevalence of community-level prevalence of C-sections. In BDHS 2022, women from communities with a high prevalence of C-section are 11.68 times more likely to have a C-section than women from communities with a low prevalence of C-section (OR=11.679 with 95% CI = 8.706, 15.668). These findings indicate a high influence of the peer group on deciding on a C-section. The regional variation was found less important determinant for C-sections in Bangladesh. Dhaka showed a higher likelihood of having a C-section than the reference group (Barishal) for BDHS 2011 and 2014. In BDHS 2017, the lowest impact of the division was observed for Chittagong. Compared to the Barishal division, women in Chittagong are 50% less likely to have had a C-section during the last birth. A surprising finding was observed for women’s place of residence; rural women were 2.6 times more likely to have had a C-section in the last birth compared to urban women in BDHS 2011 (OR = 2.673, 95% CI = 1.94, 3.68). A higher likelihood of C-sections is observed for rural women in BDHS 2014, 2017−18, and 2022 as well.

Age showed a significant impact on C-sections for the age group 30–34 in BDHS 2011 and 2014. The likelihood of having a C-section increases with age in BDHS 2011; however, different patterns are observed for the other three BDHSs. In BDHS-2011, women aged 30–34 are 2.32 times more likely (OR = 2.32, with 95% CI = 1.21, 4.45) to have a C-section in the last birth than women in the reference group (aged 15–19). In parallel to that, those who have experienced teen pregnancy (during their first childbirth) are 26% less likely (OR = 0.743 with 95% CI = 0.583, 0.947) to have a C-section in the last birth at BDHS 2022. After controlling for the effect of other variables, birth order is found to be a significant determinant of C-sections at BDHS 2014. First-order births are found to have a high likelihood of being a cesarean birth in our findings. Second-order births are 34% less likely (OR = 0.668 with 95% CI = 0.48, 0.94) to have a C-section, and for third or higher births, the likelihood is almost 45% less (OR = 0.555 with 95% CI = 0.35, 0.89) than that of the reference category (first-order birth).

The number of ANC was found to be a highly significant determinant of having a C-section in BDHS 2017−18 (Table 2). Except for BDHS-2011, the likelihood of having a C-section increases with adequate ANC visits. At BDHS 2017−18, women who had 4 or more ANC visits are 58.5% more likely (OR = 1.585 with 95% CI = 1.26, 2) to have C-sections in their last birth than women who had 3 or fewer ANC visits. This study found the second major significant association between the place of delivery (public or private facilities) and the occurrence of C-sections. Those who had their last childbirth in a private facility are 8.16 times more likely (OR = 8.160 with 95% CI = 6.658, 10.0) to have a C-section compared to births that occurred in public facilities in BDHS 2022. At BDHS 2014 women who went to private facilities were 4.729 times more likely (OR = 4.279 with 95% CI = 3.36, 5.45) and at BDHS 2017−18, they were 7.95 times more likely (OR = 7.975 with 95% CI = 6.35, 10.02) to have C-section in last birth compared to the reference group (have last birth in public facilities).

Among other individual-level variables, the likelihood of having a C-section is higher for women with secondary or higher education in BDHS 2011 and 2014. A similar pattern is observed for women whose husbands have secondary or higher-level education in the same surveys. Non-Muslim women have a lower likelihood of having a C-section in the last birth compared to Muslim women in BDHS 2011 and 2017−18. Almost similar findings were obtained for mass media exposure. In BDHS 2017−18, women who are exposed to mass media are 30% more likely (OR = 1.300 with 95% CI = 0.99, 1.71) to have a C-section in the last birth compared to those women who were unexposed to mass media. We considered only one family-level variable in our study, which is the wealth quintile. Considering the poorest group as the reference category, for all four of these DHSs, the likelihood of having a C-section increases with higher wealth classes except for the poor group in BDHS 2014.

From the findings of both bivariate and multivariate analyses, it is evident that the prevalence of C-sections has a sharp increasing trend in private facilities. The prevalence of C-sections in institutional deliveries, based on the types of endorsement (public or private), is detailed in Table 3, which provides the percentages of C-sections among all deliveries. Government facilities such as district hospitals, Maternal and Child Welfare Center, Upazilla Health Complex, and Family Welfare Center are found to have a decreasing trend of C-section over time. On the other hand, private hospitals and clinics show an increasing trend over time, rising from 75.9% C-section in BDHS 2011 to 84.7% in BDHS 2022.

**Table 3:** Distribution of C-sections among women according to different types of facilities in Bangladesh (BDHS 2011, 2014, 2017−18 & 2022).

Endorsement type	Place of delivery	BDHS 2011	BDHS 2014	BDHS 2017−18	BDHS 2022
Public	Government-Specialized & Medical College Hospitals	65.5	57.9	64.0	62
	District hospitals	47.2	45.1	43.2	46.0
	Maternal and Child Welfare Center	47.2	43.8	40.9	42.1
	Upazilla health complex and family welfare center	28.7	15.2	14.4	12.3
	Other public sectors	0.0	0.0	60.0	33.3
Private	Private medical college hospitals	100	–	87	63.6
	Private hospitals and clinics	75.9	80.4	83.5	84.7
	NGO static clinics	31.1	29.4	0	37.6
	Other private sectors (including NGOs)	0.0	0.0	42.9	33.3

*Note: For BDHS 2014, Private hospitals and clinics include private medical college hospitals as well.

## Discussion

Our study, using four waves of Bangladesh Demographic and Health Survey (BDHS) data, explored changes in the prevalence of C-section deliveries among Bangladeshi women aged 15–49 who had institutional deliveries. It uniquely examined the effects of community-level prevalence of C-sections, along with other social and economic factors, on the likelihood of delivering by C-section. The prevalence of institutional delivery due to promotional activities has increased from 31% to 65% between 2011 and 2022 in Bangladesh [14,33]. This study reported that the prevalence of C-section deliveries for all three groups: all births (of any birth order), first births, and health center (HC) based deliveries, has increased alarmingly, which are 2.2, 2.7, and 4.6 times respectively higher than the WHO recommended maximum threshold of C-section (15%) for any country [45]. The increased prevalence of C-sections in different settings was also reported in previous studies conducted in Bangladesh [15,16,33,34,36–39,46,47]. This reflects the misuse of limited health and financial resources and causes severe threats to mothers’ and newborns’ health [48–50]. An improved condition of women’s education and households’ wealth status, provision of C-sections at private and public health facilities located even union and upazila levels, and various maternal and child health-related programs might be the contributing factors to increasing the population-based C-section [15,16,25,38,51].

To the best of our knowledge, this study is the first attempt to specifically demonstrate the adverse effects of community-level prevalence of C-sections in the last birth, along with other community factors. The prevalence of C-section deliveries increased substantially with the rising prevalence of community-level C-sections. Women from communities with a higher prevalence of C-sections are more likely to opt for C-section deliveries. In contrast, an earlier cross-country study reported that the likelihood of C-sections was reduced for women who shared reproductive health information with relatives and friends [52]. This pattern should be a major concern because the health risk of mothers and neonates rises with increases the unnecessary C-sections [50]. Thus, excessive C-sections pose an additional burden for Bangladesh’s health systems to enhance the coverage of safe motherhood practices and achieve the targets of the Sustainable Development Goals for reducing mothers and neonate mortality [53]. These findings suggest a reflection of community pressure to have a C-section rather than medical necessity [3].

An increasing trend was noted among women from highly illiterate communities. Thus, the rise in C-sections is observed not only among those with higher education but also among less educated groups, likely due to perceived community pressure. However, the significant drop in the BDHS 2022 data warrants further analysis to understand the underlying reasons. The greater odds of rural women compared to urban women having C-sections, as shown in our study, also justify this. This tendency is plausibly linked with a greater availability of C-section services both in public and private hospitals and clinics almost at all tiers of administrative units, promotional activities of HC-based delivery care, and profit motives of health practitioners without increasing knowledge about the health consequences of C-sections.

In line with previous studies, our study also finds regional variations in C-section delivery rates with some rise and fall over the years [15,36,38,51]. However, C-section rates were found consistently higher in Dhaka, Khulna, and Rajshahi divisions and lower in Chittagong, Rangpur, and Sylhet divisions which were also identified as hot-spots and cold-spots of C-section deliveries respectively in Bangladesh [36,38]. Regional variation in C-sections is likely to be due to socio-cultural attributes and religious practices [38].

This study finds a greater likelihood of C-sections with the increased age of mothers except for women aged 40 and above over the years. Previous studies consistently documented more C-section deliveries among women from higher age groups [15,25,54–56]. However, the variations in C-sections by age group are likely to be diluted over time with the greater availability of C-section provision even in rural Bangladesh, and the increased accessibility of women, particularly those who are younger, to education and healthcare facilities [57–59].

Our study like the past studies reported that women with greater education, wealth, and educated husbands delivered births more through C-sections [15,16,33,34,36,38]. One possible reason for such a trend is that women who are educated and wealthy have better accessibility and affordability to private health facilities for maternal healthcare, and a greater ability to make decisions about their health [60]. Educated women also have a greater possibility of having educated husbands and being members of wealthy families. Moreover, women in Bangladesh nowadays prefer C-sections to vaginal deliveries to avoid labor pain and other consequences [21,51,56].

C-section section delivery is often found negatively correlated with women’s age at first birth and their birth order. The likelihood of C-sections was found higher among women who had teenage pregnancy and with higher birth orders. Studies conducted in South and Southeast Asia including Bangladesh also documented a comparable and significant reverse relationship between higher birth order and C-section deliveries [16,30]. Additionally, the decreased rates of C-sections with the increased age at first birth and higher birth order are likely to be linked with the couple’s greater excitement and greater concern for the first birth and babies at an early age [61,62].

This study likewise the results of past studies reported that C-section deliveries were associated positively over the years with increased ANC visits and HC-based delivery care. [15,16,33,34,36,37] Women with more ANC were more likely to have HC-based delivery, and the higher use of health facilities for delivery care increased their likelihood of delivering birth by C-section [46]. While women in Bangladesh prefer private health facilities to public health facilities for multiple causes including the unavailability of specific services, lack of awareness, unpleasant setting, and high out-of-pocket costs for antenatal and delivery care despite their greater availability and accessibility, the profit-driven private health facilities taking this opportunity contribute to the accumulative C-section deliveries without having proper health reasons [25]. Working as well as non-Muslim women were at a lower risk of having C-section deliveries over the period except for the year 2011. Past studies in Bangladesh also have shown comparable results as working women endure time constraints due to their employment, which limits their utilization of antenatal care [56,63]. While non-Muslim women’s greater risk of delivering birth by C-section is associated with their greater use of antenatal and HC-based care, the lower risk of Muslim women is linked with their cultural attributes, high parity, and previous practice of home-based delivery [61,62].

This study also found a greater risk of delivering birth via C-section among women who were exposed to mass media. However, this finding was not significant in 2014. Women’s exposure to mass media like television, newspapers, different apps of social media, and radio has increased nowadays in Bangladesh with increased ownership of mobile phones, access to electricity, internet, and the availability of mass media options, and that exposure has created awareness about maternal care options [15,25]. A cross-country study’s results which are comparable to our study reported that the consistent use of mass media options and maintaining social networks have a positive impact on the risks of C-section delivery [52].

### Strengths and limitations

One of the major strengths of this study is the use of four waves of nationally representative cross-sectional data including the recent one to examine the determinants of C-section. This study to our knowledge for the first time identified the effects of community-level prevalence of C-sections and the literature on C-sections among those who only had HC-based delivery. Another important strength is that this study compared the changes in estimates of C-section predictors over the survey years.

Despite having some strengths, this study also has some limitations. First, this study used the wealth index as a proxy for socioeconomic inequality due to the unavailability of household-level income and expenditure data in the BDHS. Second, the cross-sectional nature of the BDHS data limited our ability to establish causal relationships. Third, although our study focused on social determinants of C-section delivery, we were constrained to the variables available in the BDHS datasets. As such, other potentially relevant social and geographical factors—such as women’s decision-making autonomy, exposure to social media, gender-based violence, household exposure to natural disasters, or access to nearby health facilities—were not included due to data limitations and the scope of the analysis and should be explored in future research. Fourth, this study did not account for clinical or physiological indications for C-section delivery, as the primary focus was on social and contextual determinants.

## Conclusions

In view of the increasing prevalence of HC-based delivery, both in public and private hospitals, and the growing numbers of potential mothers due to the changing age structure in Bangladesh, the implementation and evaluation of policy measures to address the higher rates of C-sections among those who had HC-based delivery are critically important. Recognizing the robust effects of the community prevalence of C-sections on the subsequent C-section in the case of the HC-based delivery, a greater awareness program about the adversity of unnecessary C-sections is needed for rural and urban potential mothers since their conception. Counting the adverse effects of greater ANC and HC-based delivery on increased C-sections, promotional and motivational efforts are needed for medical doctors and nurses to counsel potential mothers, particularly during ANC visits, about the health consequences of mothers and newborns and encourage them to go for normal deliveries. In viewing the effects of socioeconomic status on C-section deliveries and women’s greater use of multiple media, social campaigns for raising awareness via social and electronic media would also be an effective policy option to reduce the prevalence of unnecessary C-sections.

## Supporting information

Table A1 Relative changes in C-section prevalence by background characteristics across BDHS survey years (2011–2017-18), using 2022 as the reference year.
(PDF)
