# Supplementary material for: Does community pressure matter in cesarean deliveries in Bangladesh? An analysis of nationally representative surveys
Source: PLoS One. 2025 Aug 19;20(8):e0328162. doi: 10.1371/journal.pone.0328162 (PMC12364313; doi:10.1371/journal.pone.0328162)
Supplement: Table A1 — Relative changes in C-section prevalence by background characteristics across BDHS survey years (2011–2017-18), using 2022 as the reference year. (PDF) [file pone.0328162.s001.pdf]

Appendix

Table A1 presents the relative prevalence of C-section deliveries across different background characteristics using BDHS survey data from 2011, 2014, and 2017–18, with 2022 assumed to represent the peak level and set as the reference year (100). Percentages indicate the relative prevalence in earlier years compared to 2022. P-values reflect the statistical significance of changes over time.

Table A1. Relative Changes in C-section Prevalence by Background Characteristics across BDHS Survey Years (2011–2017-18), Using 2022 as the Reference Year.

Background variables	BDHS wave			p-value
	2011 (%)	2014 (%)	2017-18 (%)	
**Community-level Prevalence of C-sections**				
Low (25 or less)	138.0	121.7	112.0	$p<0.001$
Moderate (25-50)	113.7	107.8	112.2	$p<0.001$
High (More than 50)	88.4	97.3	96.3	$p<0.001$
**Community-level Illiteracy**				
Low (25 or less)	89.7	88.1	85.4	$p=0.004$
Moderate (25-50)	87.5	90.4	99.3	$p<0.001$
High (More than 50)	110.0	102.5	106.3	$p<0.001$
**Divisions***				
Barisal	84.3	84.0	90.8	$p<0.001$
Chittagong	104.5	102.4	113.0	$p<0.001$
Dhaka	88.8	95.8	100.3	$p<0.001$
Khulna	73.0	77.5	88.2	$p<0.001$
Rajshahi	75.3	75.0	89.0	$p<0.001$
Rangpur	62.2	72.7	88.4	$p<0.001$
Sylhet	108.9	95.6	110.5	$p<0.001$
**Place of Residence**				
Urban	66.7	91.2	79.2	$p<0.001$
Rural	68.7	97.4	96.3	$p=0.01$
**Age of Mothers**				
15-19	79.9	81.5	86.7	$p<0.001$
20-24	100.0	95.8	96.1	$p=0.004$
25-29	99.7	94.9	97.0	$p=0.002$
30-34	98.4	98.0	98.7	$p=0.708$
35-39	83.6	89.3	94.1	$p=0.708$
40+	90.8	92.0	94.4	$p=0.708$
**Age at First Birth**				
Not Teen Pregnancy	100.0	100.0	100.0	$p<0.001$
Teen Pregnancy	76.2	80.8	85.7	$p<0.001$
**Birth Order**				
1	106.5	105.4	102.5	$p=0.062$
2	99.7	99.2	100.0	$p=0.003$
≥3	89.9	91.8	92.8	$p<0.001$

**Number of ANC Visits**				
≤3	87.4	80.8	85.5	p=0.003
≥4	97.4	99.2	99.1	p<0.001
**Place of Delivery**				
Public Facilities	61.0	51.5	51.7	p<0.001
Private Facilities	77.0	88.7	92.0	p<0.001
**Education of Mother**				
Primary or Lower	74.8	69.0	79.8	p<0.001
Secondary or Higher	100	100	100	p<0.001
**Employment Status of Mother**				
Unemployed	91.5	95.3	99.6	p=0.253
Employed	91.6	95.1	97.0	p=0.169
**Husband's Education Level**				
Primary or Lower	72.0	73.6	75.6	p<0.001
Secondary or Higher	100.0	100.0	100.0	p<0.001
**Religion**				
Muslim	104.4	105.1	101.5	p=0.373
Others	101.7	101.1	98.1	p=0.452
**Mass Media Exposure**				
Unexposed	74.4	80.7	78.0	p<0.001
Exposed	91.7	94.5	93.9	p<0.001
**Wealth Quintile**				
Poorest	39.3	45.8	56.5	p<0.001
Poorer	81.6	80.0	92.6	p<0.001
Middle	83.3	89.0	92.6	p<0.001
Richer	84.2	94.5	98.3	p<0.001
Richest	100.0	100.0	100.0	p<0.001

Note: “Division”, Mymensingh merged with Dhaka for BDHS 2017-18 to harmonize the data with BDHS 2014 & 2011.
